# Revision of three camaenid and one bradybaenid species (Gastropoda, Stylommatophora) from China based on morphological and molecular data, with description of a new bradybaenid subspecies from Inner Mongolia, China

**DOI:** 10.3897/zookeys.372.6581

**Published:** 2014-01-22

**Authors:** Pei Wang, Qiong Xiao, Wei-Chuan Zhou, Chung-Chi Hwang

**Affiliations:** 1 Key Laboratory of Molluscan Quarantine and Identification of AQSIQ, Fujian Entry-Exit Inspection & Quarantine Bureau, Fuzhou, Fujian 350001, China; 2 Department of Life Sciences, National University of Kaohsiung, No.700, Kaohsiung University Road, Nan-Tzu District, Kaohsiung 81148, Taiwan

**Keywords:** *Satsuma*, *Ganesella*, *Bradybaena*, revision, new subspecies

## Abstract

We have revised the taxonomy of three camaenid and one bradybaenid species from China and described one new subspecies of the genus *Bradybaena* (Family Bradybaenidae) from Inner Mongolia, China. The genitalia of three *Satsuma* (Family Camaenidae) species *S. mellea stenozona* (Moellendorff, 1884), *S. meridionalis* (Moellendorff, 1884), **comb. n.** and *S. uncopila* (Heude, 1882), **comb. n.** assigned to the genus *Bradybaena* previously,lack a dart sac and mucous glands. Moreover, the molecular phylogeny has revealed close relationships between the three species and the genus *Satsuma*. Two species, *S. stenozona* (Moellendorff, 1884) from Fuzhou and *Ganesella citrina* Zilch, 1940 from Wuyi Mountain, are considered as synonymous and should be a subspecies of *S. mellea mellea* (Pfeiffer, 1866) because of the morphological and molecular similarities. Meanwhile, the other two are placed in the genus *Satsuma*: *S. meridionalis* (Moellendorff, 1884), **comb. n.** and *S. uncopila* (Heude, 1882), **comb. n.** *G. virgo* Pilsbry, 1927 differs from species of the genera *Ganesella* and *Satsuma* not only in its shell, but also in anatomical characters, such as having a dart sac and mucous gland, and lacking a flagellum. Additionally, phylogenetic analyses highly support the sister relationship with other *Bradybaena* species. Thus, placement of *G. virgo* Pilsbry, 1927 in the genus *Bradybaena* issuggested.

## Introduction

The land snail families Camaenidae and Bradybaenidae are extremely specious, and both families are difficult groups to deal with in terms of taxonomy. The camaenids occur across a wide geographical area from the northern to southern hemisphere, such as China, Japan, Taiwan, Philippines, Indonesia, New Guinea, Australasia, America (Scott 1996; Cuezzo 2003). There is no special synapomorphy which is characteristic of this group (Scott 1996). Usually, they are defined by the absence of dart sac and dart-related organs on the female genitalia (Pilsbry 1939). Morphological studies and molecular phylogeny are contradictory about the monophyly or paraphyly of the group (Scott 1996; Cuezzo 2003; Wade et al. 2007). The bradybaenids have maximal diversity in Southeast and East Asia, Northwest America and Europe, including China, Russian Far East and Siberia, Japan, Korean, Taiwan, Philippines, Indonesia (Pilsbry 1900; Zilch 1959–1960; Minato 1985; Chang and Hwang 2000; Hsieh et al. 2006). Generally, the bradybaenids are identified based on the presence of dart sac and dart-related organs, however, some studies have suggested that the absence of dart-related organs occurred in a number of lineages (Davison et al. 2005; Wade et al. 2007; Hirano et al. 2014). Moreover, certain incongruence among the morphology, taxonomy, and molecular phylogeny of the bradybaenid land snails has been found (Hirano et al. 2014). These studies indicate that traditional morphology-based systematics may largely stray from molecular phylogeny, hence, the combination of morphology, anatomy and molecular studies is quite essential in biological classification.

The genus *Ganesella* Blanford, 1863 (*sensu* Zilch 1959–1960, Gastropoda: Stylommatophora: Camaenidae) was erected for the type species *Helix capitum* Benson 1848 from India. Most of snails in the genus *Ganesella* have very small ranges. They are mainly distributed in South-east and South Asia (Tryon 1888; Pilsbry 1894; Zilch 1959–1960, 1966; Richardson 1985; Chen and Gao 1987; Azuma 1995). However, the classification of several species in *Ganesella* is still confused (Kuroda and Habe 1949; Richardson 1985; Vaught 1989; Azuma 1995; Wu 1999). Species distributed in East Asia were revised in previous taxonomic publications with a broad focus (Minato 1988; Hsieh et al. 2006; Wu et al. 2008; Schileyko 2011; Zhou et al. 2011). All eastern Asian species originally assigned to the genus *Ganesella* (from Honshu, Japan through Ryukyu to Taiwan) were subsequently transferred to the genus *Satsuma*, which was synonymous with *Coniglobus* Pilsbry & Hirase, 1906, *Luchuhadra* Kuroda & Habe, 1949 and *Pancala* Kuroda & Habe, 1949 (Minato 1988; Hsieh et al. 2006; Hwang 2011). However, there is still no consensus on the classification of species occurring in China, almost all of which are still catalogued in *Ganesella*, with the only exception *Satsuma stenozona* (Pilsbry 1894; Zhou et al. 2011).

The traditional classification of *Ganesella* relies predominantly on shell features. Purportedly characteristic features are, for instance, a thin, high, lustrous and conical shell, white to pale brown shell color, and a slightly descending body whorl (Yen 1939; Zilch 1966; Chen and Gao 1987). Most Chinese *Ganesella* species are conchologically more similar to *Satsuma* in having a conical to depressed conical shell of corneous color. Chinese species are often confused with the genera *Plectotropis* Martens, 1860, *Aegista* Albers, 1850 and *Bradybaena* Beck, 1837 of Bradybaenidae owing to the morphological similarity of shells. Clearly, our knowledge of the Chinese species remains comparatively poor (Zhou et al. 2011). In order to contribute to a better understanding of their taxonomy, three species from China currently placed in *Bradybaena* and onespecies currently placed in *Ganesella* are revised on the basis of morphological, anatomical and molecular evidence. One new subspecies of the genus *Bradybaena* from Inner Mongolia, China is described for the first time.

## Material and methods

**Figure 1.**
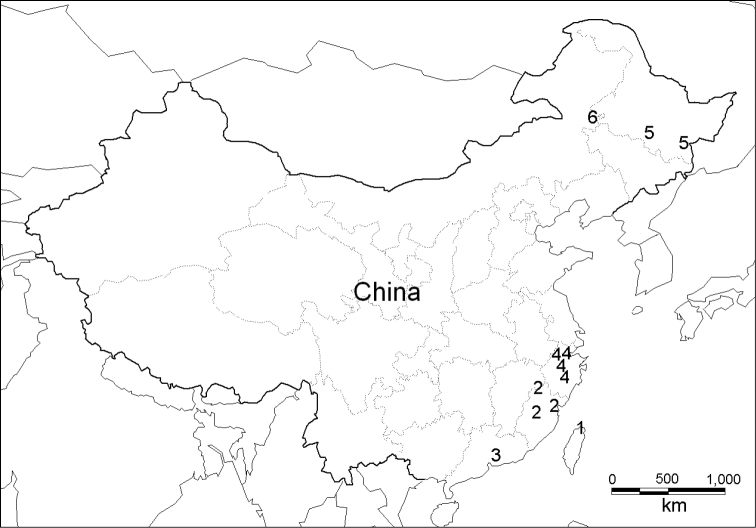
Map of sampling sites. **1** *Satsuma mellea mellea* (Pfeiffer, 1866) **2** *Satsuma mellea stenozona* (Moellendorff, 1884) **3** *Satsuma meridionalis* (Moellendorff, 1884) **4** *Satsuma uncopila* (Heude, 1882) **5** *Bradybaena virgo virgo* (Pilsbry, 1927) **6** *Bradybaena virgo mongolia* subsp. n.

**Material.** This study is based on material collected by the authors from several sites in China (Fig. 1). Live adults were drowned in water for 12–24 hours, then killed in hot water, preserved in 75% or 95% ethanol, and stored at -20°C. Samples have been deposited in the State Key Laboratory of Molluscan Quarantine and Identification, Fujian Entry-Exit Inspection & Quarantine Bureau, Fuzhou, China (FJIQBC).

Abbreviations used: IZCAS, Institute of Zoology, Chinese Academy of Science Museum, Beijing, China; SMF, Senckenberg Natural History Museum, Frankfurt am Main, Germany.

**Methods.** Shells were measured to 0.1 mm using electronic calipers. Standard shell parameters were taken following Dillon (1984). More than 15 specimens of each species were measured.

Genitalia of adult snails were dissected under a dissecting microscope (ZEISS Discovery V20). All drawings were traced with the aid of a Canon 550D digital camera. Terminology for reproductive system follows Gómez (2001). More than three specimens of each species were dissected.

Total genomic DNA was extracted from muscle tissue of foot using Qiagen DNeasy Blood & Tissue kit (Qiagen, Hilden, Germany). Polymerase chain reaction (PCR) was performed to amplify a fragment (615 bp) of the mitochondrial cytochrome c oxidase subunit I gene (*COI*) using a pair of universal primers (LCO1490: 5’-ggtcaacaaatcataaagatattgg-3’; HCO2198: 5’-taaacttcagggtgaccaaaaaatca-3’) (Folmer et al. 1994) from 16 specimens of 13 species. Short PCR reactions were performed using Takara Taq DNA polymerase (Takara, Dalian, China), with the following cycling conditions: 30 s at 94 °C, followed by 35 cycles of 10 s at 94 °C, 30 s at 45 °C, and 1 min at 72 °C. The final elongation step was continued for 10 min at 72 °C. The PCR products were analyzed by spectrophotometry and 1.0% agarose gel electrophoresis. All fragments were sequenced from both directions after purification using the BigDye Terminator Sequencing Kit (Applied Biosystems, San Francisco, CA, USA) and the ABI 3730XL Genetic Analyzer (PE Applied Biosystems). Sequence electropherograms were proof-read and aligned into contigs using BioEdit v7.0.5.3 (Hall 1999). Phylogenetic analyses were performed using 23 *COI* fragments including sequences of additional species retrieved from GenBank (Table 1). *Cornu aspersum* belonging to the family Helicidae was used as outgroup. Multiple alignment and Maximum-likelihood (ML) analysis were performed using Mega v5.0 (Tamura et al. 2011) with default settings. Model selection was done with Modeltest 3.7 (Posada and Crandall 1998). The node support values were assessed by bootstrap resampling (Felsenstein 1985) using 1000 replicates.

**Table 1.** Sample information.

Family	Sampling	Locality	Collection date	Coordinates	Accession number
Camaenidae	*Satsuma mellea stenozona* (n=2)	Gushan, Fuzhou, Fujian	2010.10	26°03'26"N, 119°24'02"E	KF765745/KF765746
	*Satsuma mellea stenozona*	Wuyi Mountain, Fujian	2010.10	27°39'02"N, 117°58'01"E	KF765744
	*Satsuma mellea mellea*	Ilan, Taiwan	1997.06	24°45'05"N, 121°36'43"E	KF765743
	*Satsuma meridionalis*	Luofushan, Guangdong	2010.11	23°16'03"N, 114°03'37"E	KF765756
	*Satsuma uncopila*	Hangzhou, Zhejiang	2011.10	30°07'04"N, 120°02'26"E	KF765758
	*Satsuma largillierti* ^†^	Japan			AB242499
	*Satsuma pekanensis* ^†^	Taiwan			EF204833
	*Satsuma nux* ^†^	Taiwan			EF057347
	*Satsuma batanica pancala* ^†^	Taiwan			AB480901
	*Satsuma nux paiwanis* ^†^	Taiwan			EF204824
	*Satsuma succincta* ^†^	Taiwan			EF204839
Bradybaenidae	*Cathaica fasciola fasciola*	Beijing	2008.10	39°59'51"N, 116°10'50"E	KF765749
	*Plectotropis yonganensis*	Yongan, Fujian	2011.03	26°03'32"N, 117°19'44"E	KF765747
	*Plectotropis brevibarbis*	Tianmu Mountain, Zhejiang	2011.05	30°20'21"N, 119°23'58"E	KF765748
	*Aegista permellita*	Leshan, Sichuan	2011.05	29°32'45"N, 103°46'16"E	KF765759
	*Bradybaena ravida*	Xiaoshan, Zhejiang	2011.05	30°10'19"N, 120°16'20"E	KF765753
	*Bradybaena similaris*	Fuzhou, Fujian	2008.08	26°09'50"N, 119°16'55"E	KF765752
	*Bradybaena sequiniana*	Badong, Hubei	2011.06	31°02'46"N, 110°22'18"E	KF765750
	*Bradybaena brevispira*	Emei Mountain, Sichuan	2011.05	29°35'28"N, 103°22'47"E	KF765755
	*Bradybaena magnaciana*	Chongqin	2011.06	29°46'21"N, 106°27'53"E	KF765754
	*Bradybaena virgo virgo*	Haerbin, Heilongjiang	2008.08	45°42'31"N, 126°38'38"E	KF765751
Helicidae	*Cornu aspersum*	France	2010.08		KF765757

†: sequence from Genbank.

## Results and discussion

### Camaenidae Pilsbry, 1895
*Satsuma* Adams, 1868

**Type species.** *Satsuma japonica* Pfeiffer, 1847, original designation.

#### Satsuma mellea stenozona

(Moellendorff, 1884)

http://species-id.net/wiki/Satsuma_mellea_stenozona

Figs 2A ; 3A ; 4A

Helix stenozona Moellendorff, 1884: 385, pl. 9, figs 5–6.
Euhadra stenozona , Pilsbry, 1890: 119, pl. 27, figs 4–5; Pilsbry, 1895: 214.
Bradybaena stenozona , Yen, 1939: 132, pl. 13, fig. 53.
Ganesella citrina Zilch, 1940: 113–118, pl. 7, fig. 4; 1966: 208, Pl. 5, fig. 26.
Ganesella stenozona , Zilch, 1966: 209, pl. 5, fig. 25.
Bradybaena (Bradybaena) stenozona , Wu, 1999: 99–100, figs 6.52–14, pl. 11B; Chen and Zhang 2004: 140–142, fig. 105.
Satsuma stenozona , Zhou et al., 2011: 52, fig. 1.

##### Type lo cality.

Fuzhou (26°5'N, 119°18'E), China.

##### Material examined.

*Bradybaena stenozona*: Fuzhou, Fujian, Lectotype (SMF 8833), paralectotype (SMF 8832); National Forest Park of Fuzhou, Fujian (May 6, 2007, 26°09'50.36"N, 119°16'55"E; FJIQBC 18220–18237); Drum Mountain of Fuzhou, Fujian (Oct. 16, 2010, 26°03'26"N, 119°24'2"E; FJIQBC 18238–18245); YuHua Hole of Jiangle, Fujian (Jun. 1, 2007, 26°41'59"N, 117°30'55"E, FJIQBC 18146–18250). *Ganesella citrina*: Guadun, Wuyi Mountain, Fujian, Holotype (SMF 47228), paratypes (SMF 47229); Wuyi Mountain, Fujian (Oct. 12, 2010, 27°39'2"N, 117°58'01"E, FJIQBC 18251–18255).

##### Shell.

Dextral,medium sized, about 14.5 mm in height, 21.0 mm in width, thin but solid, straw colored, glossy; 5 ^1^/_2_ whorls. Apex obtuse. Suture deep. Spire low conical, slowly increasing, slightly convex. Body whorl fast expanding, convex, with weakly angulated margin. Periphery bluntly angulated with red-brown peripheral band, extending from apex to columellar lip. Whorls slightly descending at the front. Surface with oblique, curved growth lines, and staggered, delicate spiral lines. Aperture diagonal and round to lunate. Peristome white, slightly expanded and reflected. Inner lip with thin callus only. Basal lip curved. Columellar lip margin slightly expanded. Umbilicus open, small.

##### Reproductive system.

Penis slender, with a short penial caecum near the penis retractor. Epiphallus as wide as penis, half as long as penis. Flagellum short, about 1/5 of length of epiphallus. Penis retractor muscle thin and long. Vas deferens short. Free oviduct moderately long, slightly inflated. Vagina short. Pedunculus of bursa copulatrix inflated at base, fusiform. Bursa copulatrix oval.

##### Ecology.

One of the collected sites, Yuhua Hole, Jiangle, Fujian belongs to a Karst land form (limestone), all others are on Danxia land forms (acidic soil). Snails generally live under rotten branches and fallen leaves in forests, and actively crawl on trees during rainy seasons. Population density is generally not high in these locations. In Fuzhou, snails become active in early April, brisk in May and June, lie dormant in the soil by the end of October; juveniles and eggs aestivate during winter. Newly hatched snails will grow into adult in 7–8 months, then mate and spawn, about 100–200 eggs at once. Eggs are large, 1.5–2.0 mm in diameter.

##### Remark.

This species has been placed in *Bradybaena* for a long time. Based on a study of the types, Zilch (1966) transferred it to the genus *Ganesella*, assuming a close relationship with *Ganesella mellea mellea* (Pfeiffer, 1866) (=*Satsuma mellea*) from Taiwan and *Ganesella citrina* Zilch, 1940 from Wuyi Mountain. However, his classification was not refuted subsequently (Wu 1999; Chen and Zhang 2004). Eventually, this species was classified as a member of the genus *Satsuma* by Zhou et al. (2011) for a lack of accessory sac as well as mucous gland, but the authors didn’t provide any molecular evidence.

In the present study, the phylogenetic analyses based on *COI* showed close phylogenetic relationships and short genetic distances between specimens identified as *Satsuma stenozona*, *Ganesella citrina* and *Satsuma mellea* (Fig. 5). The shell features of *Satsuma stenozona* from Fuzhou and *Ganesella citrina* from Wuyi Mountain do not reveal obvious differences. The differences mentioned by Zilch (1940), such as the shell dimensions and color bands, are mere variations between individuals and populations. The molecular phylogeny also indicated that *Satsuma stenozona* and *Ganesella citrina* were sister taxa. Therefore, we consider *Ganesella citrina* a synonym of *Satsuma stenozona*. *Satsuma mellea* and *Satsuma stenozona* may be considered as geographical races of the same species for the rather low amounts of morphological and molecular difference (Chang 1981; Zhou et al. 2011). Hence, we classified *Satsuma stenozona* as a subspecies of *Satsuma mellea*.

**Figure 2.**
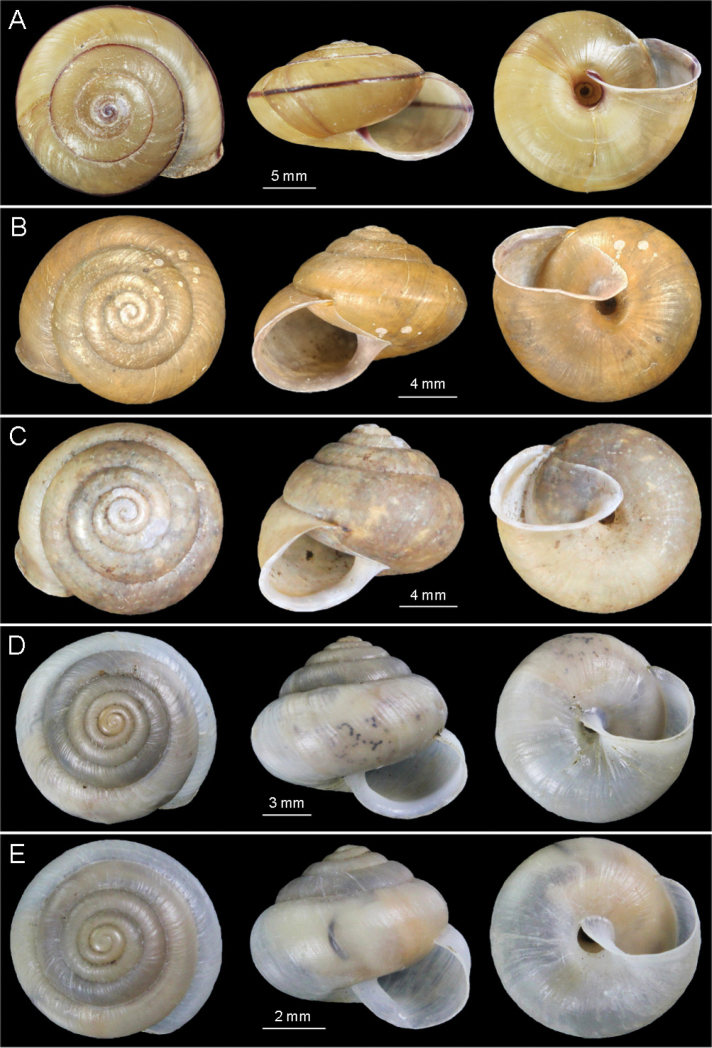
Photographs of shells. **A** *Satsuma mellea stenozona* (Moellendorff, 1884) (FJIQBC 18221, Fuzhou, China) **B** *Satsuma meridionalis* (Moellendorff, 1884) (FJIQBC 18415, Guangdong, China) **C** *Satsuma uncopila* (Heude, 1882) (FJIQBC 18417, Hangzhou, China) **D** *Bradybaena virgo virgo* (Pilsbry, 1927) (FJIQBC 18432, Haerbin, China) **E** *Bradybaena virgo mongolia* subsp. n. (Holotype, FJIQBC 18466, Inner Mongolia, China).

**Figure 3.**
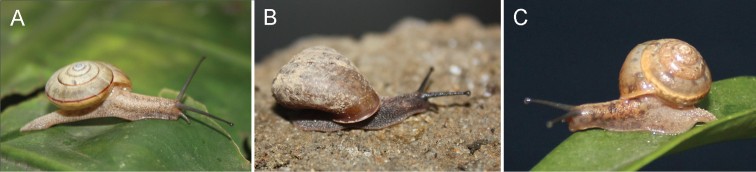
Ecological photographs of snails. **A** *Satsuma mellea stenozona* (Moellendorff, 1884) (National Forest Park, Fuzhou, Fujian) **B** *Satsuma meridionalis* (Moellendorff, 1884) (Luofu Mountain, Guangdong) **C** *Satsuma uncopila* (Heude, 1882) (Lingshan Hole, Hangzhou, Zhejiang).

**Figure 4.**
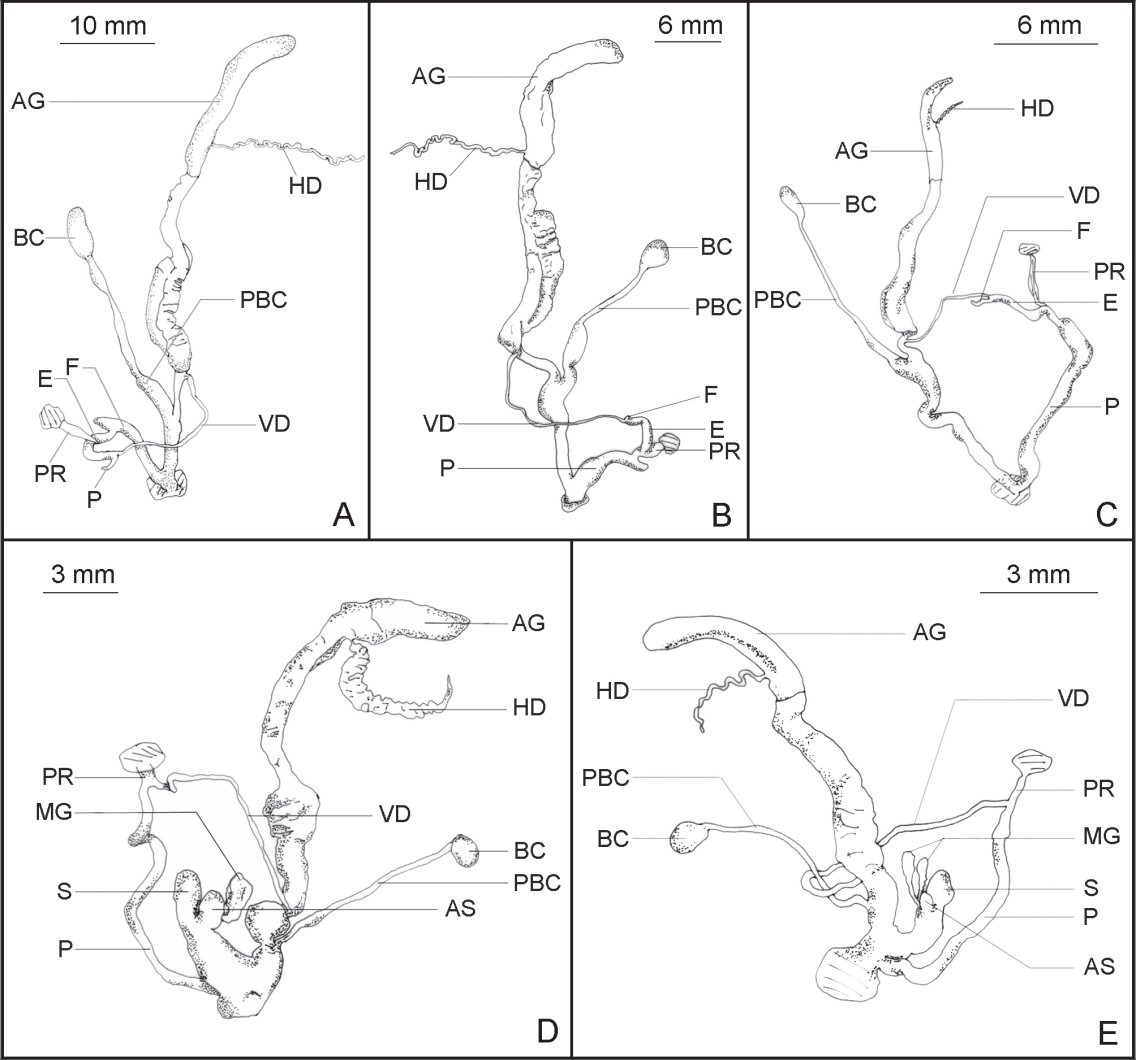
Reproductive system. **A** *Satsuma mellea stenozona* (Moellendorff, 1884) (FJIQBC 18237, Fuzhou, China) **B** *Satsuma meridionalis* (Moellendorff, 1884) (FJIQBC 18416, Guangdong, China) **C** *Satsuma uncopila* (Heude, 1882) (FJIQBC 18423, Hangzhou, China) **D** *Bradybaena virgo virgo* (Pilsbry, 1927) (FJIQBC 18462, Haerbin, China) **E** *Bradybaena virgo mongolia* subsp. n. (Paratype, FJIQBC 18471, Inner Mongolia, China).

**Figure 5.**
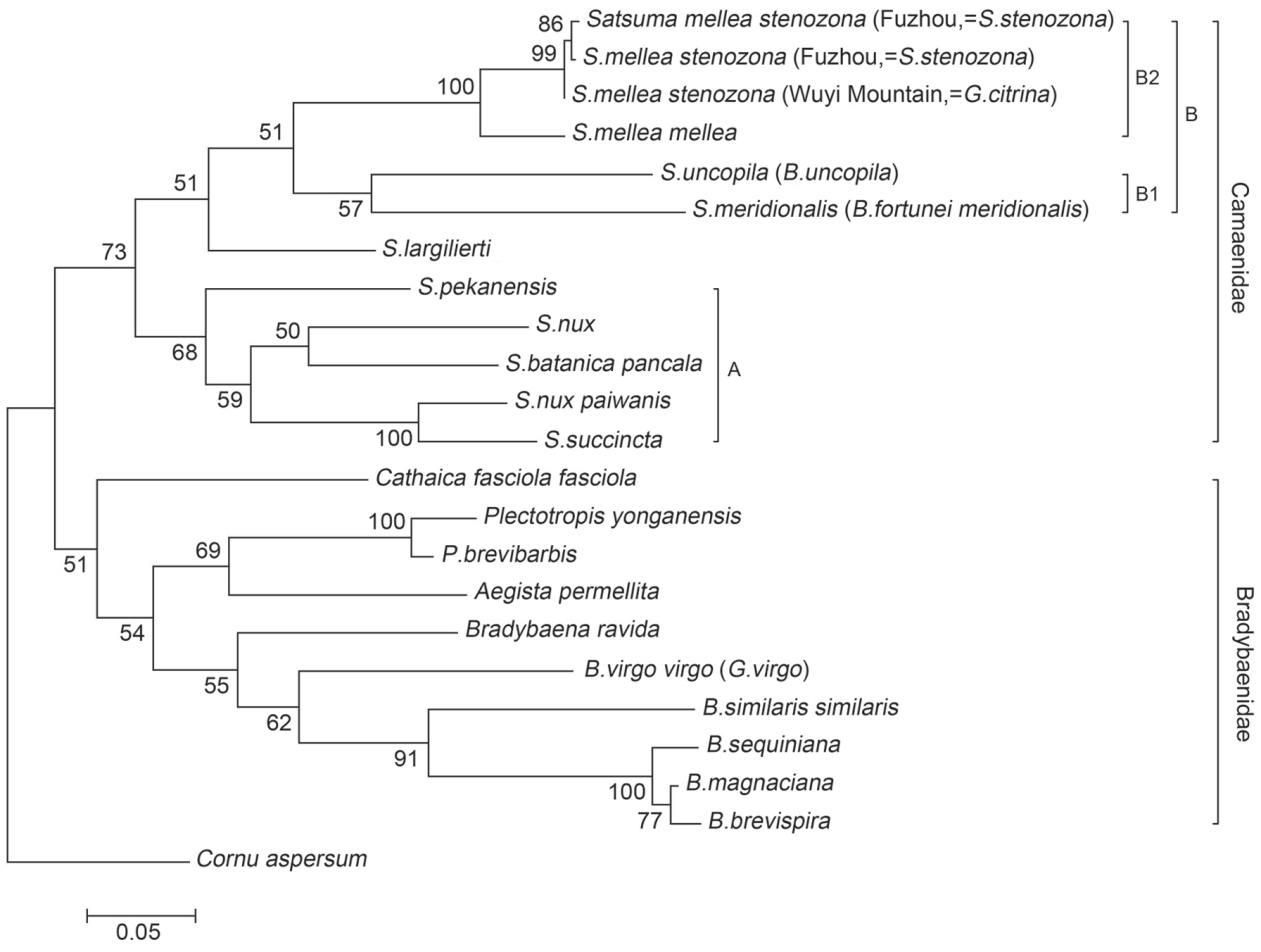
Phylogenetic tree inferred by maximum likelihood (ML) method based on *COI* gene. The tree is rooted with *Cornu aspersum* (Müller, 1774). Numbers near the nodes represent bootstrap values.

#### Satsuma meridionalis

(Moellendorff, 1884) comb. n.

http://species-id.net/wiki/Satsuma_meridionalis

Figs 2B ; 3B ; 4B

Helix fortunei var. *meridionalis* Moellendorff, 1884: 327, pl. 7, fig. 5.
Helix (Dorcasia) fortunei var. *meridionalis*, Tryon, 1887: 208, pl. 47, fig. 51.
Helix (Euhadra) fortunei var. *meridionalis*, Pilsbry, 1890: pl. 15, figs 69–70.
Eulota (Eulota) fortunei var. *meridionalis*, Pilsbry, 1895: 204.
Bradybaena fortunei meridionalis Yen, 1939: 134, pl. 13, fig. 66.
Bradybaena fortunei submeridionalis Zilch, 1951: 86; Zilch, 1968: 183.
Bradybaena (Bradybaena) fortunei Richardson, 1983: 27; Wu, 1999: 83–84, pl. 7B; Chen and Zhang 2004: 145–146, fig. 111.

##### Type locality.

Luofu Mountain, Guangdong (23°16'03"N, 114°03'37"E), China.

##### Materials examined.

Luofu Mountain, Lectotype (SMF 9155), paralectotype (SMF 9156); Luofu Mountain, Guangdong (Nov. 3, 2010, 23°16'03"N, 114°03'37"E; FJIQBC 18407–18416).

##### Shell.

Sinistral,medium sized; about 11.0 mm in height, 15.2 mm in width, thin but solid, yellowish-brown in color, depressed conic; 5 ^1^/_2_ whorls. Surface with dense growth lines and weak spiral lines. Spire slightly low conical, slowly increasing, slightly convex. Body whorl fast expanding, quite convex. With slight, slender and dull red band on periphery of body whorl for most specimens. Periphery bluntly angulated. Aperture descending and elliptical. Peristome thin, sharp, slightly reflected. Inner lip with thin callus. Columellar lip short, reflected, slightly covering umbilicus. Umbilicus deep, round, and about 1/5 of width of shell.

##### Reproductive system.

Penis thick and short, with an expanded base. Penial caecum short. Epiphallus slender, about 2/3 of length of penis. Flagellum short and small, about 1/10 of length of epiphallus. Penis retractor muscle thick and wide. Vas deferens long and slender. Oviduct thin. Vagina longer than penis, expanding at posterior end. Pedunculus of bursa copulatrix expanding at base. Bursa copulatrix oval.

##### Ecology.

The species usually lives in the wet bushes and grass near farmland, especially on limestone cliffs and in cracks with more humus, or under rotten branches and fallen leaves; occasionally within human settlements. This snail is sensitive to low temperature, aestivates from November to March. Animals often feed on all kinds of crops, especially tender shoot and leaf.

##### Remark.

Originallyit was described as variety of *Helix fortunei* (Pfeiffer, 1850) for its uniformly yellowish-corneous color and globularly conic shell shape. Subsequently, Yen (1939) treated it as the subspecies *Bradybaena fortunei meridionalis*. However, Chen and Zhang (2004) rejected the subspecies arrangement and syonymized the name *meriodionalis* with *Bradybaena fortunei*. In the current study, we dissected the genitalia of the species, revealing lack of dart sac and mucus gland. Therefore, the species is now recognized as *Satsuma meridionalis* according to shell features, characters of genitalia and the molecular phylogeny (Fig. 5). We are unable to address the systematic relationships with *Bradybaena (Bradybaena) fortunei* from Shanghai for the lack of suitable material.

#### Satsuma uncopila

(Heude, 1882) comb. n.

http://species-id.net/wiki/Satsuma_uncopila

Figs 2C ; 3C ; 4C

Helix uncopila Heude, 1882: 41, pl.16, fig. 16; Moellendorff, 1884: 327.
Helix (Dorcasia) uncopila , Tryon, 1887: 208, pl. 47, fig. 56.
Eulota uncopila , Pilsbry, 1895: 204.
Eulota (Eulota) uncopila , Gude, 1902: 7.
Bradybaena uncopila , Yen, 1939: 134, pl. 13, fig. 67; Zilch, 1968: 187.
Bradybaena (Bradybaena) uncopila , Richardson, 1983: 39; Wu, 1999: 101, pl. 11D; Chen and Zhang 2004: 147–148, fig. 111.

##### Type locality.

The Yangtze valley, China.

##### Material examined.

Lingshan Hole, Hangzhou, Zhejiang (Oct. 5, 2011, 30°07'04"N, 120°02'26"E; FJIQBC 18417–18423); Tianmu Mountain, Zhejiang (May 6, 2011, 30°20'21"N, 119°23'58"E; FJIQBC 18424–18245); Yaolin fairyland, Tonglu, Zhejiang (May 25, 2008, 29°53'08"N, 119°37'09"E, FJIQBC 18426–18428); Shuanglong Hole, Jinhua, Zhejing (May 2, 2009, 29°12'23"N, 119°37'09"E, FJIQBC 18429–18431).

##### Shell.

Sinistral,medium sized, about 11.5 mm in height, 16.8 mm in width, thin, fawn colored, conical. Whorls 5. Surface with short and diagonal growth lines, and weak spiral lines. Spire higher. Body whorl fast increasing, expanding but not descending at the front. Periphery smooth, not convex. Apex obtuse. Suture deep. Aperture elliptical. Peristome slightly thickened, reflected, white, occasionally reddish-brown. Columellar lip reflected, slightly covering umbilicus. Umbilicus narrow and small.

##### Reproductive system.

Penis long and thicker. Epiphallus slender, about 1/4 of length of penis. Flagellum short, thin, about 1/3 of length of epiphallus. Penis retractor muscle thin, moderately long. Vas deferens short, slender. Oviduct thin, short. Vagina long, gradually expanding towards posterior end. Pedunculus of bursa copulatrix slender, expanding at base. Bursa copulatrix oval.

##### Ecology.

The snail ordinarily lives in the wet bushes and grass on hills, especially in places that are rich in humus, under rotten branches and fallen leaves; also frequently found on limestone cliffs and in cracks.

##### Remark.

This species has previously been placed in the family Bradybaenidae, but it is here transferred to the Camaenidae for the lack of dart sac and mucous gland. Following our phylogenetic analyses, we assign it to the genus *Satsuma* (Fig. 5).

#### Bradybaena virgo virgo

(Pilsbry, 1927)

http://species-id.net/wiki/Bradybaena_virgo_virgo

Figs 2D ; 4D

Ganesella virgo Pilsbry, 1927: 461, pl. 35, f. 7.7a.
Ganesella murensis , Cockerell, 1926: 227.
Fruticicola virgo , Kuroda, 1941: 27–28.
Bradybaena (Virginihelix) virgo , Kuroda, 1949, 64, f. 30.
Bradybaena (Virginihelix) virgo , Habe, 1956, f. 1.
Ganesella virgo , Chen and Gao 1987: 108, f. 138.

##### Type locality.

Uiju, North Pyongan, North Korea.

##### Material examined.

Plant Park of Haerbin, Heilongjiang (Aug. 26, 2008, 45°42'31"N, 126°38'38"E; FJIQBC 18432–18462); Suburb of Jidong, Heilongjiang (Aug. 29, 2008, 45°14'57”, 131°09'01"E; FJIQBC 18463–18465).

##### Shell.

Dextral,medium sized, about 12.0 mm in height, 13.5 mm in width, thin but solid, semitranslucent, glossy, spherical. Whorls 6–6 ^1^/_2_. Apex sharp. Suture deep. Spire conical, slowly increasing, convex. Body whorl fast expanding, convex, about 3/4 of height of shell. Surface pale white or yellow, with dense and clear growth lines, and unambiguous spiral lines. Aperture descending at the front, elliptical. Peristome reflected. Columellar lip reflected, partly covering umbilicus. Umbilicus small.

##### Reproductive system.

Penis long, slender, moderately wide. Flagellum absent. Penis retractor muscle thin, wide and short. Vas deferens short and slender. Oviduct short and inflated. Dart sac large, oval, with one smaller accessory sac. One mucus gland, kinkled. Pedunculus of bursa copulatrix slender, short. Bursa copulatrix oval.

##### Ecology.

The snail often lives on damp pastures, especially near ditch, or in grass.

##### Remark.

This species is the first intermediate host of *Eurytrema pancreaticum*, a parasite of humans and livestock (Tang et al. 1979; Tang et al. 1980; Gu et al. 1990). Recently, several studies on bionomics and control measures of the snail have been published. However, the taxonomic status has been unclear (Zhu et al. 1989; 1990). Originally assigned to the Camaenidae, *Ganesella virgo* has subsequently been transferred to the Bradybaenidae based on anatomical and shell features by Kuroda (1941, 1949) and Habe (1956). This treatment, however, has been widely neglected by Chinese workers. In the present study, we dissected several specimens collected in Haerbin and Jidong, Heilongjiang, and found that anatomical characters were in concordance with the description of Kuroda (1941, 1949) and Habe (1956). In addition, the molecular phylogeny confirmed close relationships with other species in *Bradybaena*. Thus, *Ganesella virgo* is correctly placed in *Bradybaena*.

#### Bradybaena virgo mongolia

Wang & Zhou subsp. n.

http://zoobank.org/58D99BDE-0764-49DE-9954-B3C5EE7A024C

http://species-id.net/wiki/Bradybaena_virgo_mongolia

Fig. 2E ; 4E

##### Etymology.

For the type locality, adjective.

##### Holotype.

(FJIQBC 18466) Shell height 6.5 mm, width 7.0 mm, height of aperture 3.5 mm, width of aperture 3.6 mm, October 5, 1982, collected from the type locality.

##### Paratypes 14 specimens.

(FJIQBC 18467–18471) and (IZCAS TM 126010–126018) Shell height 5.5–7.0 (6.4±0.40) mm, width 6.4–7.5 (7.1±0.25) mm, height of aperture 3.2–3.6 (3.4±0.13) mm, width of aperture 3.3–3.7 (3.5±0.16) mm, October 5, 1982, collected from the type locality.

##### Type locality.

The grassland of Zhalaiteqi, Inner Mongolia, China (46°43'59"N, 123°19'20"E).

##### Description.

Dextral, small sized, thin but solid, semi-translucent, lustrous, globular. Whorls 6 on average, with conical spire. Shell light yellow or white in color, with some dense and well-developed growth lines. Spiral lines on body whorl weak. Apex sharp. Suture deep. Last whorl constricted, expanded towards the base, convex, comprising about 3/4 of shell high. Aperture elliptical. Peristome reflected, with white, thickened callus inside. Inner lip and columellar lip reflected, partly covering umbilicus. Umbilicus narrow, deep.

##### Reproductive system.

Penis long. Flagellum absent. Penis retractor muscle slender, moderately long. Vas deferens moderately long. Oviduct short and thick. Vagina short. Dart sac inflated, thick. Accessory sac small. Two mucus glands. Pedunculus of bursa copulatrix slender, but not long. Bursa copulatrix oval.

##### Ecology.

The snail usually lives on damp pastures, especially in tall and dense grass, i.e., *Achnatherum splendens*. However, it is difficult to collect this animal because of serious grassland degradation in Inner Mongolia.

##### Remark.

The new subspecies resembles *Bradybaena virgo virgo* in morphology, but the two subspecies can be differentiated by the following characteristics: (1) The subspecies *mongolia* has a smaller shell (shell height 5.5–7.0 mm, width 6.4–7.5 mm) than *Bradybaena virgo virgo* (shell height 12.0 mm, width 13.5 mm), (2) it has two mucus glands instead of one in the nominate form, and (3) its umbilicus is wider (about 1/9 of the shell width) than in the nominate form (about 1/12 of the shell width).

### Molecular analysis

Twenty-three partial sequences of *COI* were analyzed. The aligned sequences contained no indels and were deposited in GenBank (Table 1). The molecular phylogeny was based on the analysis of 615 unambiguously aligned nucleotide sites, of which 253 were variable and 233 were parsimony informative. According to the Akaike information criterion, the general time reversible model with a proportion of invariable sites and a gamma shaped distribution of rates across sites (GTR + I + G) was the best-fitting model of sequence evolution. All other settings for ML analysis were kept as default.

The ML tree (Fig. 5) presented two major clades corresponding to the families Camaenidae and Bradybaenidae, respectively. *Bradybaena virgo virgo* originally classified in *Ganesella* belonged to a clade of taxa in *Bradybaena*, and this agreed with the anatomical result. Thus the placement of this species in *Bradybaena* is suggested.

The clade of taxa in the family Camaenidae contained three subclades, *Satsuma* species from Taiwan in group A, *Satsuma largillierti* from Japan and species from southeast China and north Taiwan in group B. In addition, species in group B were divided into two subgroups, including subgroup B1 with sinistral shell and subgroup B2 with dextral shell, and this is consistent with the study on the reproductive system above. Therefore, the two species *Bradybaena meridionalis* from Luofushan, Guangdong and *Bradybaena uncopila* from Hangzhou, Zhejiang in subgroup B1, which were originally classified in Bradybaenidae, should be assigned to the family Camaenidae. On the other hand, *Satsuma stenozona* from Fuzhou and *Ganesella citrina* from Wuyi Mountain in subgroup B2 appeared monophyletic. There were low amounts of morphological difference between species from Fujian and *Satsuma mellea* from Taiwan with geographic isolation. In view of the above, the two taxa from Fujian are revised as a subspecies of *Satsuma mellea* (Fig. 5).

In the present study, three camaenids and one bradybaenid from China were revised on the base of morphological and molecular characters, but the systematics of the remaining Chinese species in the superfamily Camaenoidea are still problematic. Camaenids and bradybaenids may be more complex than we have previously suspected. In the future, more samplings will be required to resolve this problem.

## Supplementary Material

XML Treatment for Satsuma mellea stenozona

XML Treatment for Satsuma meridionalis

XML Treatment for Satsuma uncopila

XML Treatment for Bradybaena virgo virgo

XML Treatment for Bradybaena virgo mongolia
